# Performance and application analysis of ANFIS artificial intelligence for pressure prediction of nanofluid convective flow in a heated pipe

**DOI:** 10.1038/s41598-020-79628-w

**Published:** 2021-01-13

**Authors:** Meisam Babanezhad, Iman Behroyan, Ali Taghvaie Nakhjiri, Azam Marjani, Saeed Shirazian

**Affiliations:** 1 Institute of Research and Development, Duy Tan University, Da Nang, 550000 Vietnam; 2 Faculty of Electrical and Electronic Engineering, Duy Tan University, Da Nang, 550000 Vietnam; 3 Department of Artificial Intelligence, Shunderman Industrial Strategy Co., Tehran, Iran; 4 Department of Computational Fluid Dynamics, Shunderman Industrial Strategy Co., Tehran, Iran; 5 Faculty of Mechanical and Energy Engineering, Shahid Beheshti University, Tehran, Iran; 6 Department of Petroleum and Chemical Engineering, Science and Research Branch, Islamic Azad University, Tehran, Iran; 7 Department for Management of Science and Technology Development, Ton Duc Thang University, Ho Chi Minh City, Vietnam; 8 Faculty of Applied Sciences, Ton Duc Thang University, Ho Chi Minh City, Vietnam; 9 Laboratory of Computational Modeling of Drugs, South Ural State University, 76 Lenin Prospekt, Chelyabinsk, Russia 454080

**Keywords:** Engineering, Mathematics and computing

## Abstract

Heat transfer augmentation of the nanofluids is still an attractive concept for researchers due to rising demands for designing efficient heat transfer fluids. However, the pressure loss arisen from the suspension of nanoparticles in liquid is known as a drawback for developing such novel fluids. Therefore, prediction of the nanofluid pressure, especially in internal flows, has been focused on studies. Computational fluid dynamics (CFD) is a commonly used approach for such a prediction of fluid flow. The CFD tools are perfect and precise in prediction of the fluid flow parameters. But they might be time-consuming and expensive, especially for complex models such as 3-dimension modeling and turbulent flow. In addition, the CFD could just predict the pressure, and it is disabled for finding the relationship of such variables. This study is intended to show the performance of the artificial intelligence (AI) algorithm as an auxiliary method for cooperation with the CFD. The turbulent flow of Cu/water nanofluid warming up in a pipe is considered as a sample of a physical phenomenon. The AI algorithm learns the CFD results. Then, the relation between the CFD results is discovered by the AI algorithm. For this purpose, the adaptive network-based fuzzy inference system (ANFIS) is adopted as AI tool. The intelligence condition of the ANFIS is checked by benchmarking the CFD results. The paper outcomes indicated that the ANFIS intelligence is met by employing *gauss2mf* in the model as the membership function and x, y, and z coordinates, the nanoparticle volume fraction, and the temperature as the inputs. The pressure predicted by the ANFIS at this condition is the same as that predicted by the CFD. The artificial intelligence of ANFIS could find the relation of the nanofluid pressure to the nanoparticle fraction and the temperature. The CFD simulation took much more time (90–110 min) than the total time of the learning and the prediction of the ANFIS (369 s). The CFD modeling was done on a workstation computer, while the ANFIS method was run on a normal desktop.

## Introduction

The improvement of the thermophysical properties of the operating fluids is known as a way for the efficiency augmentation of the heat exchangers being employed in different industrial applications such as oil and gas industry. Using nanofluids (NFs) has been introduced for decades as such an improvement in heat transfer where these fluids have significant applications^1^. The dynamic viscosity and the thermal conductivity of the base fluids are influenced by the suspension of particular nanoparticles with designed shape and size. There are a number of studies have shown the conductivity and viscosity increase of the NFs by the nanoparticle concentrations^2–4^. Mahbubul et al.^3^ reviewed the experimental investigations about the nanofluids viscosity. They reported that viscosity is enhanced by the increment of volume concentration and is deteriorated by increasing the temperature. Abulhassan et al.^5^ carried out an investigation on the evaluation of the viscosity of Titania nanotubes ethylene glycol/water-based nanofluid. They believe that the nanofluid’s thermophysical properties must be assessed based on the nanoparticle concentration and the base fluid temperature.

According to the literature pertaining to NFs, although the heat transfer possessed the potential of enhancement by the NFs, the more pressure is lost by the nanoparticles fraction. Moghanlou et al.^6^ conducted a systematic experimental-based work on the nanofluid heat transfer in a mini channel. They also considered the pressure drop in NFs. They revealed the increment of heat transfer by 9.30 and 4.56% for 0.5% volume fraction of Al_2_O_3_–water and TiO_2_–water, respectively. More pressure loss than water was also reported for both nanofluids. In^7^, a similar study was carried out on the nanofluid convective flow in channels. According to their results, heat transfer as well as pressure loss rose by improving the nanoparticles volume fraction in the NF. So, the prediction of the changes in the nanofluid pressures in internal flows such as pipes and channels are important as a case for investigation. The computational fluid dynamics (CFD) tools have been widely employed by studies^8–10^. For example, Behroyan et al.^11–13^ performed a comprehensive numerical investigation on different two-phase and single-phase CFD for modeling convective flow of NFs. They found that the single-phase model considering the Brownian model in thermal conductivity and the Lagrangian two-phase model shows the closest predictions to the experimental data. Garoosi et al.^1^ used the CFD tool for modeling a heat exchanger working with nanofluids. Bhatti et al.^14^ mathematically modeled MHD nanofluid flow through a porous cylinder pondering the chemical reaction. It was shown that the chemical reaction parameter possessed a substantial influence on increasing the nanoparticle concentration profile. A similar study on MHD nanofluid flow through horizontal parallel plates was carried out and reported in^15^. It was corroborated by their study that as the magnetic parameter and nanoparticle concentration increase, the nanofluid speed declines, while the temperature profile increases. Reddy et al.^16^ modeled the thermal and hydrodynamic parameters of Williamson nanofluid flow over a stretching sheet. The numerical results showed that by enhancing the magnetic field, the velocity profiles decrease. However, the temperature and nanoparticle concentration profiles rise.

The CFD methods could effectively and accurately predict the pressure of the nanofluid flows in different circumstances. However, it could be expensive and time-consuming, especially in 3-dimension modeling and turbulent flow. In addition, CFD modeling alone could predict the pressure. An auxiliary method is required to find the relationship of the pressure to its effective parameters, including nanoparticle fraction and the temperature. Recently the Artificial Intelligence (AI) algorithm has shown some helps to tackle the CFD modeling difficulties in complicated cases^17–23^. The CFD results could be learned by the AI algorithm. Then, the AI algorithm could understand the logical behavior of the targeted variables with the changes in the boundary conditions, fluid properties, etc. For example, Babanezhad et al.^24^ used the AI algorithm of the adaptive network in combination with the fuzzy inference system (ANFIS) for prediction of gas/liquid interaction of two-phase bubbly flow in a column reactor. They reported^17^ high compatibility between the ANFIS and CFD outputs. The authors employed the ANFIS simulations in the combination with the Lagrangian CFD approach for particle tracking in a cavity. They claimed that using the ANFIS method could cause faster computational activities. However, there is not any data to compare the calculation time of the CFD and the ANFIS. This approach is in the first step of investigations. There is not any study to use the AI algorithms for developing the relationship between the CFD results. Besides, a comparison between the computational performance of the CFD and the AI algorithms does not exist. For filling such gaps, this research is intended to establish the adaptive network-based fuzzy inference system (ANFIS) as an auxiliary tool to collaborate with the CFD approach. For this purpose, the intelligence of the ANFIS is investigated and verified by the validated CFD results. The relationship of pressure with the nanoparticles fraction and the fluid temperature is founded. The computational performance and requirements of the CFD are compared with the ANFIS.

## Methodology

### CFD technique

In this investigation, ANSYS FLUENT V.16.1 software as a commercial package was appropriately applied on the basis of finite volume technique (FVT) to build the mechanistic model and run the simulations. In terms of the employed boundary conditions of model, constant heat flux was postulated for the walls, and uniform velocity and temperature were assumed for the inlet of the considered geometry.

According to the above-mentioned employed boundary conditions, 3-D governing equations are derived for the momentum/heat transfers as below^25–27^:

Continuity equation:

1 $$\nabla \cdot \left( {\rho_{f} V} \right) = 0$$

Momentum equation:

2 $$\nabla .\left({\rho }_{f}VV\right)=-{\nabla }_{p}+\nabla .\tau$$

Energy equation:

3 $$\nabla .\left({\rho }_{f}V{C}_{p.f}T\right)=\nabla .\left({k}_{f}\nabla T-{C}_{p.f{\rho }_{f}}\overline{VT}\right)$$

The $k-\varepsilon$ turbulence model is known as a well-known model applied in this study to compute the energy dissipation rate $(\varepsilon )$, eddy viscosity, and kinetic energy $(k)$ of turbulency as follows^12,13,25,28^:

4 $$\nabla .(\rho kV) = \nabla .[(\frac{{\mu_{t} }}{{\sigma_{k} }})\nabla (k)] + G_{k} - \rho \varepsilon$$

5 $$\nabla .(\rho \varepsilon V) = \nabla .[\frac{{\mu_{t} }}{{\sigma_{\varepsilon } }}\nabla \varepsilon ] + \frac{\varepsilon }{k}(C_{1\varepsilon } G_{k} - C_{2\varepsilon } \rho \varepsilon )$$

6 $$G_{k} = \mu_{t} (\nabla V + (\nabla V)^{T} ), \, \mu_{t} = \rho C_{\mu } \frac{{k^{2} }}{\varepsilon }$$

7 $$C_{\mu } = 0.09,\sigma_{k} = 1.00,\sigma_{\varepsilon } = 1.30,C_{1\varepsilon } = 1.44,C_{2\varepsilon } = 1.92$$

8 $$k_{t} = \frac{{C_{p} \mu_{t} }}{{\Pr_{t} }}, \, \Pr_{t} = 0.8$$

### Nanofluid properties

In this work, a nanofluid constitutes of water as base fluid and copper (Cu) nanoparticles is simulated. All correlations being used in this study for calculation of the Cu/water NF properties are given in Table 1. User Defined Function (UDF) codes were developed for temperature-dependent conductivity (Brownian motion of nanoparticle) and it was added to ANSYS-FLUENT CFD code to complete the simulations.

**Table 1:** Cu/water properties used in the simulations ^12,13^.

Properties	Equation
Density	$\rho_{eff} = (1 - \varphi )\rho_{f} + \varphi \rho_{p}$
Heat capacity	$c_{p,eff} = \frac{{(1 - \varphi )(\rho c_{p} )_{f} + \varphi (\rho c_{p} )_{p} }}{{(1 - \varphi )\rho_{f} + \varphi \rho_{p} }}$
Viscosity^31^	$\mu_{nf} = (1 + 7.3\phi + 123\phi^{2} )\mu_{f}$
Thermal conductivity	${k}_{eff}/{k}_{bf}=1+{\left(\frac{{k}_{p}}{{k}_{bf}}\frac{{d}_{bf}}{{d}_{p}}\frac{\phi }{1-\phi }\right)}(1+\mathrm{25,000}\frac{{u}_{b}{d}_{p}}{{k}_{bf}})$ Brownian motion velocity of nanoparticles $:{u}_{b}=\frac{2{K}_{B} T}{\pi {\mu }_{bf}{{d}_{p}}^{2}}$

### Numerical method and grid test

**Figure 1:**
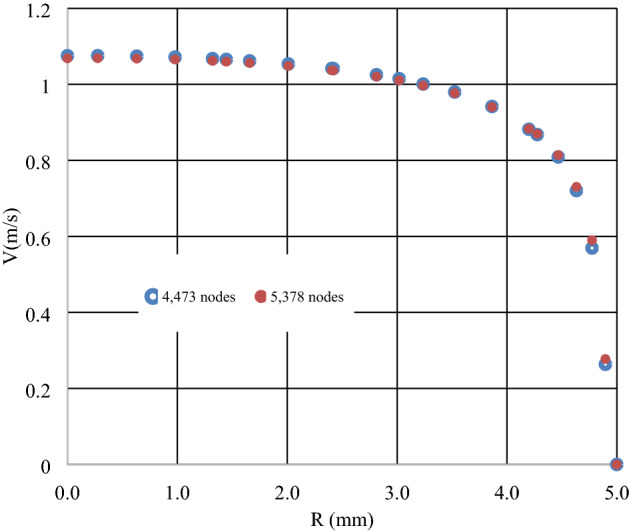
Velocity profile of the Cu/water nanofluid inside the pipe.

This study has employed the numerical procedures of the Ansys Fluent CFD package of, V19. The Fluent works according to the finite volume technique (FVT). As discretization procedure, the 2nd order upwind technique is employed in the computations. SIMPLE coupling method was adopted to link velocity and pressure. For the meshing process, design modeller toolbox in Ansys has been applied to construct the computational mesh. The mesh independence test was implemented for two sets of grid arrangements (i.e., 4473 nodes, and 5378 nodes). Comparing the mesh configurations, in terms of the velocity profile, the relative deviations were less than 0.05% (Fig. 1). Hence, the first grid case has been opted for the current investigation.

### Validation test

For validation of the CFD outcomes, the estimated Nusselt values in this work are compared with the achieved measured data from the literature (Table 2). The results show a good agreement with a maximum of 12% deviation.

**Table 2:** Validation test of CFD^32,33^.

Nanoparticle fraction (%)	Nu _exp_	Nu_CFD_
0.5	72.74	63.74
1	80.82	75.74
1.5	88.28	82.04
2	94.66	88.15

### ANFIS

Soft computing approaches or intelligent algorithms have extensive utilization to predict the physical phenomena in industries and academia. Such techniques have the capability of learning physics and simulating the phenomena via its language. Its structure can be established based on the needed demands and evaluations. The current study deals with a learning framework for understanding the current data and defining an ANFIS pattern according to the information for predicting the non-present data in the considered specific data package. We created a novel set up data for estimation in the final phase of code development. Former papers ^29,30^ developed this phase for predicting the fluid properties.

ANFIS method is explained as the learning stage of fuzzy system decisions and neural networks. Presently, in ANFIS pattern, the 1^st^ order Sugeno was implemented to represent 32 fuzzy rules:

9 $${\text{if x is A}}_{{1}} {\text{and y is C}}_{{1}} \quad \varpi_{1} = \kappa_{1} x + q_{1} y + r_{1}$$

10 $${\text{if x is A}}_{{2}} {\text{and y is C}}_{{2}} \quad \quad \varpi_{2} = \kappa_{2} x + q_{2} y + r_{2}$$

11 $$\ldots {\text{and if x is A}}_{{{32}}} {\text{and y is C}}_{{{32}}} \quad \quad \varpi_{32} = \kappa_{32} x + q_{32} y + r_{32}$$

**Figure 2:**
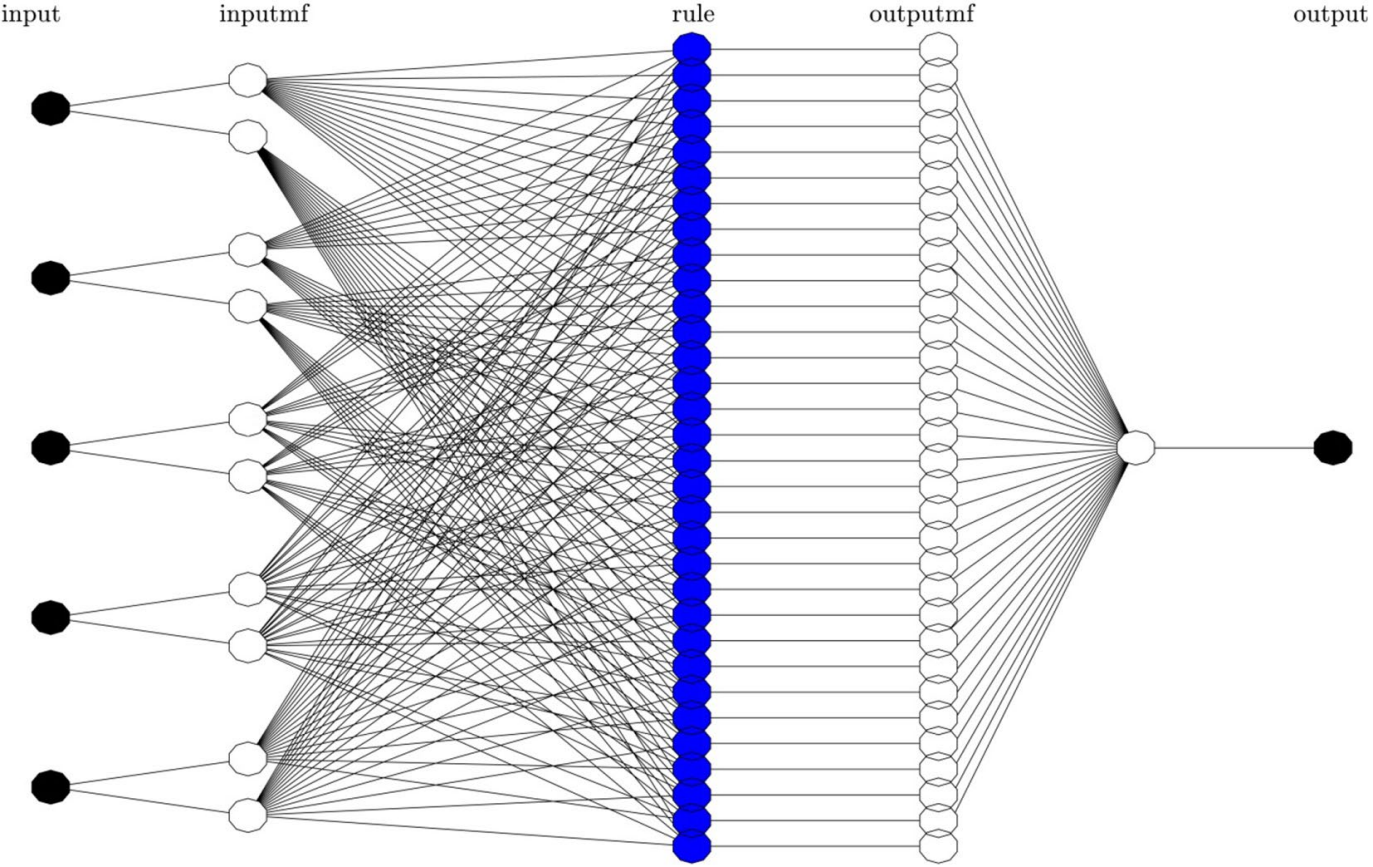
ANFIS structure with five inputs and two membership functions for each input.

Figure 2 represents the logical system for the Sugeno model within the ANFIS structure. The ANFIS structure is included the number of membership functions (MFs) and rules in the inputs, the hidden layer, and the output. No. of clusters equals 2 for each input in this model. So, 2 membership functions (MF) are considered for each input and each function is in relation to the other functions of the other inputs. So, totally the number of rules and membership functions for the hidden layer and output is equal to 32 (i.e., 2^5^). According to Fig. 2, ANFIS topology is comprised of 5 strands in which outputs of each node are calculated. Considering the output of each layer in the ANFIS network, the following layers functions of this network could be explained:

It is approximately corresponding to determine the neural nodes in the ANFIS model with similar layer. The output function of ${\Gamma }^{th}$ node in layer one is denoted by the $Q\left( {l,{\Gamma }} \right)$ as:

12 $$\begin{gathered} Q_{{1,{\Gamma }}} = \mu_{{A_{1} {\Gamma }}} \left( x \right),{\Gamma } = 1,2 \hfill \\ Q_{{1,{\Gamma }}} = \mu_{{A_{2} {\Gamma } - 2}} \left( x \right),{\Gamma } = 3,4 \hfill \\ \ldots {\text{ and}}\,\,Q_{{1,{\Gamma }}} = \mu_{{A_{5} {\Gamma } - 8}} \left( x \right),{\Gamma } = 9,10 \hfill \\ \end{gathered}$$

Layer one: The neural nodes are of adaptive type, and the bell function is utilized due to the considerable robustness for generalization of nonlinear parameters to estimate the interface as:

13 $$\mu A\left( x \right) = \frac{1}{{1 + (\frac{{x - c_{{\Gamma }} }}{{a_{{\Gamma }} }})^{{2b_{{\Gamma }} }} }}$$

Furthermore, ${a}_{\Gamma },{b}_{\Gamma },{c}_{\Gamma }$ denote a group of variables. This type of function has a changing role in terms of the number of adjustments for different parameters. Therefore, this function possesses the potential to cover some operational types for fuzzy functions of group A.

Layer 2: The function output can be expressed as:

14 $$Q_{{2,{\Gamma }}} = \omega_{{\Gamma }} = \mu_{{A_{1} {\Gamma }}} \left( X \right)\mu_{{A_{2} {\Gamma }}} \left( Y \right)\mu_{{A_{3} {\Gamma }}} \left( Z \right)\mu_{{A_{4} {\Gamma }}} \left( \varphi \right)\mu_{{A_{5} {\Gamma }}} \left( T \right),{\Gamma } = 1,2, \ldots ,32$$

Layer 3: In this layer of ANFIS, the smart junctions cannot be altered, and the output can be obtained as:

15 $$Q_{{3,{\Gamma }}} = \overline{\omega }_{{\Gamma }} = \frac{{\omega_{{\Gamma }} }}{{\omega_{1} + \omega_{2} + \omega_{3} + \omega_{4} + \omega_{5} }},{\Gamma } = 1,2, \ldots ,32$$

The outputs of this layer are known as normalized discharge abilities for greater accessibility.

Layer 4: The function may be expressed as:

16 $$Q_{{4,{\Gamma }}} = \overline{\omega }_{{\Gamma }} \varpi_{i} = \overline{\omega }_{{\Gamma }} \left( {n_{{\Gamma }} X \times o_{{\Gamma }} Y \times p_{{\Gamma }} Z \times q_{{\Gamma }} \varphi \times r_{{\Gamma }} T \times s_{{\Gamma }} } \right)$$

$n_{{\Gamma }} ,o_{{\Gamma }} ,p_{{\Gamma }} ,q_{{\Gamma }} ,r_{{\Gamma }} ,s_{{\Gamma }}$ denote a set of parameters within the node.

Layer 5: total value of outputs are calculated by this neural mesh as:

17 $$Q_{{5,{\Gamma }}} = \mathop \sum \limits_{{\Gamma }} \overline{\omega }_{{\Gamma }} \varpi_{{\Gamma }} = \frac{{\mathop \sum \nolimits_{{\Gamma }} \omega_{{\Gamma }} \varpi_{{\Gamma }} }}{{\mathop \sum \nolimits_{{\Gamma }} \omega_{{\Gamma }} }}$$

Operative inputs are established to reach level 4. To re-colonize the resulting factors, the least-squares are predicted. The error value has a backward movement in the backward pass. Using the premise factors, a gradient is descended.

## Results and discussion

The use of the nanofluids instead of their base fluids is a deal between the heat transfer and the pressure loss. The solid particles dispersion inside the base fluids could lead to the change of the thermo-physical specifications like viscosity and thermal conductivity. Although the thermal conductivity increment could be considered as an advantage, the increase of the dynamic viscosity is a drawback. The more thermal conductivity, the more heat transfer. But the more viscosity, the more pressure is lost along the pipe, and the more pumping power is needed. So, the pressure of nanofluids in wall-bounded flow has a crucial role. The nanofluids pressure is significantly related to the viscosity. The viscosity, in turn, depends on the nanoparticles fraction and the fluid temperature. The CFD method could predict the flow characteristics, but this approach lacks the ability to find the connection between the fluid flow parameters. The potential of Artificial Intelligence (AI) algorithms could be used for developing a relation between the pressure of the nanofluid and its temperature and particle fraction in any position of the flow domain.

The present paper tries to predict the pressure of a Cu/water flow in a pipe under constant wall heat transfer, and turbulent regime. The prediction, at first, is done by the CFD tool. Table 3 illustrates the simulation cases, assumptions, and the CFD method considered in this paper. The CFD outcomes are validated with the experimental data from the literature. Then, 75% of the CFD results are learned by the ANFIS, as the AI algorithm. The ANFIS predicts the whole CFD results, and the precision of the ANFIS predictions would be checked by comparison with the CFD. The intelligence of the ANFIS method is also tested by tuning different factors (x, y, z, nanoparticle content, and temperature) and various MF types.

**Table 3:** CFD information table.

Simulation cases	Turbulent flow of Cu/water NF in a heated pipe Nanoparticle volume fractions: 0.3–2% Pipe wall heat flux: 85,000 w/m^2^ Inlet velocity: 0.91 m/s Inlet temperature: 300 K
Assumptions	Homogeneous dispersion of the particles in the base fluid No thermal, hydrodynamic and chemical interactions between the Nanoparticles and the base fluid Considering Brownian effect Average nanoparticle diameter: 100 nm
CFD method	Finite Volume Method (FVM)

**Figure 3:**
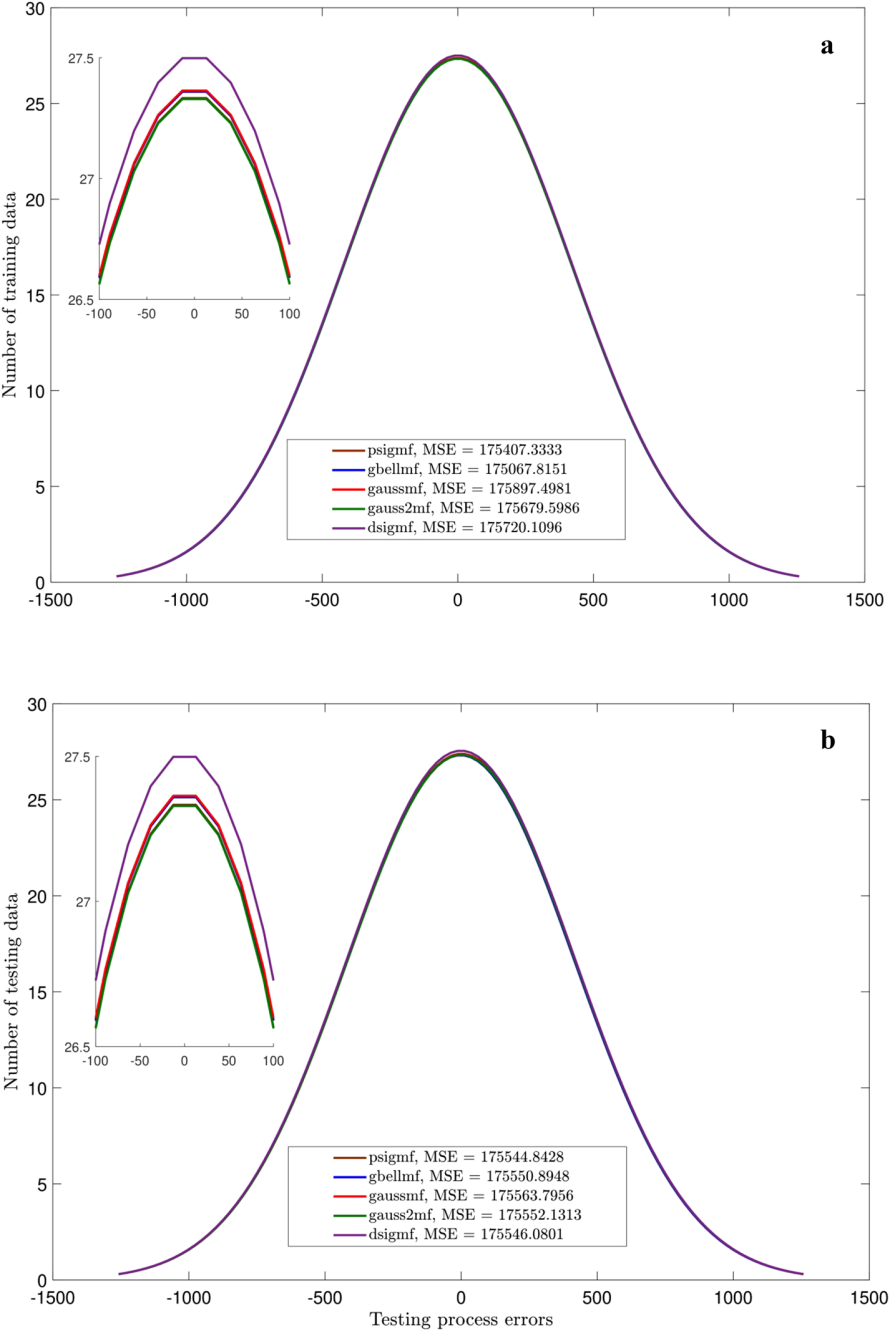
(**a**) Errors of training for different MF. Number of inputs = 2. (**b**) Errors of testing for different MF. Number of inputs = 2.

**Figure 4:**
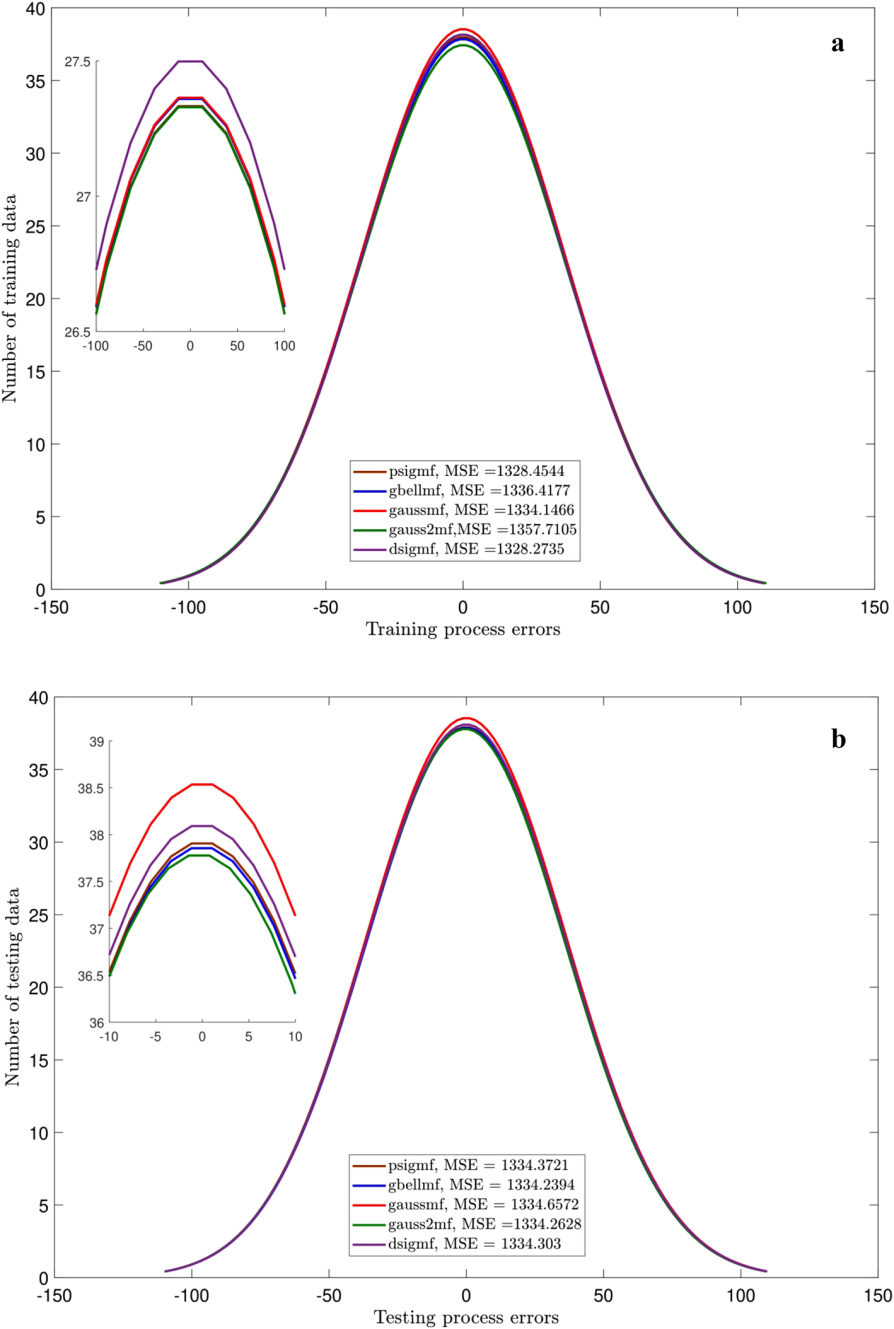
(**a**) Errors of training for different MF. Number of inputs = 3. (**b**) Errors of testing for different MF. Number of inputs = 3.

**Figure 5:**
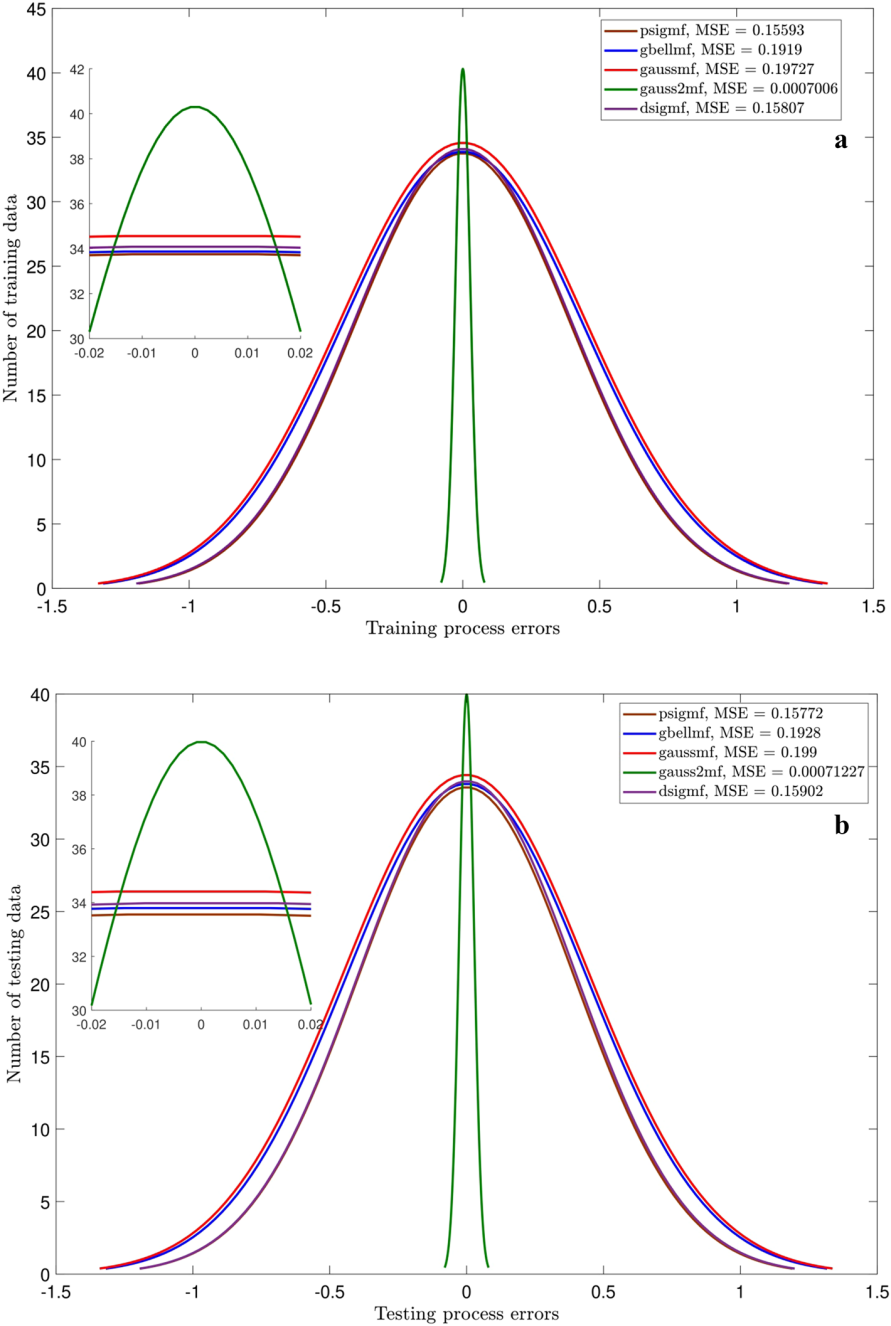
(**a**) Errors of training for different type of membership function when number of inputs is four. (**b**) Errors of testing for different type of membership function when number of inputs is four.

**Figure 6:**
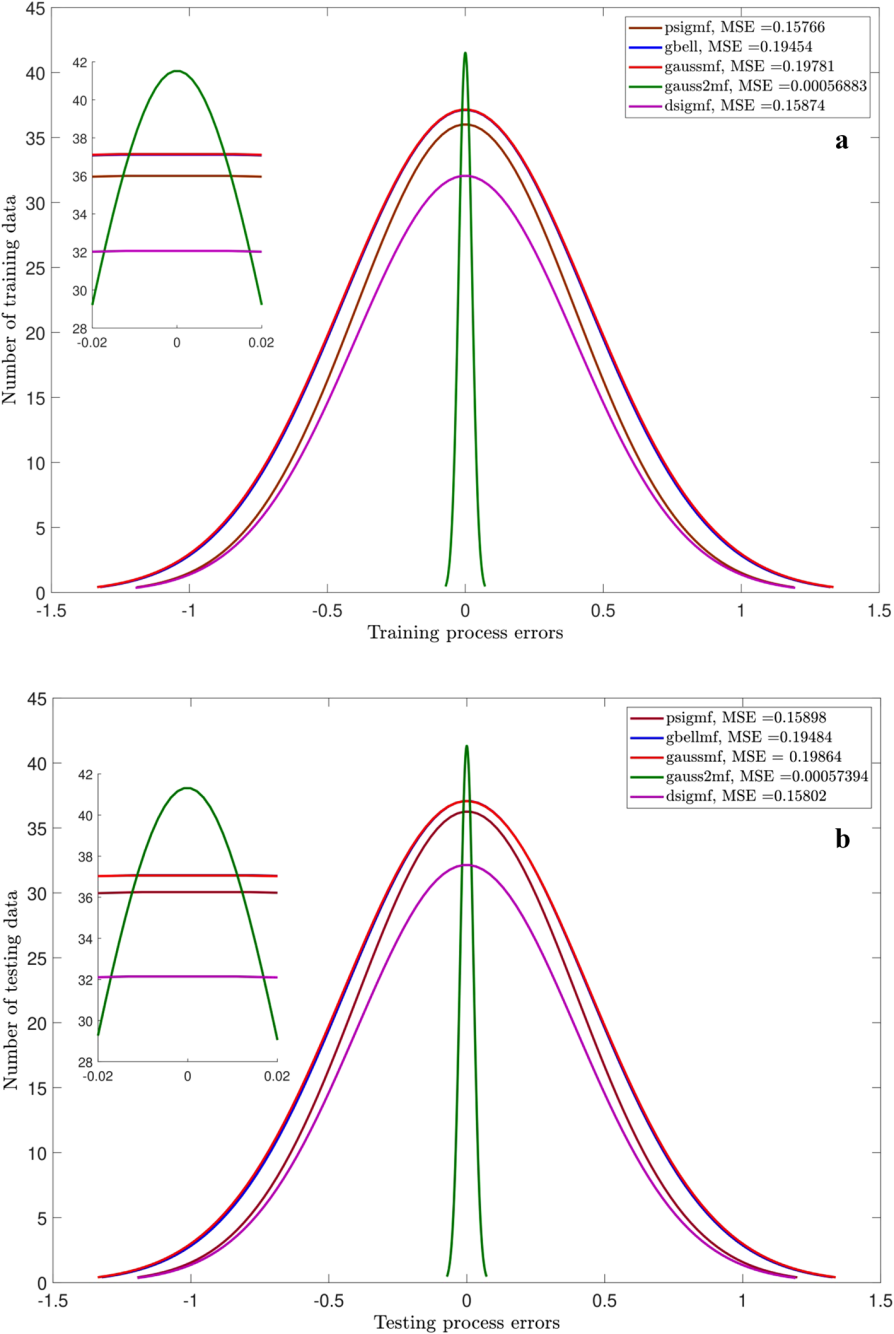
(**a**) Errors of training for different type of membership function when number of inputs is five. (**b**) Errors of testing for different type of membership function when number of inputs is five.

Figures 3,4,5 and 6 show the intelligence examinations of the ANFIS. The results are shown by the histogram curves (i.e., normal distribution of error) and their corresponding mean standard error (MSE) and correlation coefficient (R) values. According to Figs. 3a,b,4a,b, no variations are seen in histogram curves by changing the types of MF. For 2 inputs, the errors are distributed between ± 1000, while this distribution is between ± 100 for 3 inputs. As the number of inputs enhances from 2 to 3, the MSE declines from 175,550 to 1334, while the R gets closer to 1 (~ 0.996). Albeit, according to Figs. 5a,b, considering one more input (i.e., nanoparticle volume fraction), the domain of the error distribution decreases significantly (i.e., a maximum of ± 1.5), the MSE is decreased below 0.2, while the R value is still around unity. In this case, histogram curves are also sensitive to the type of membership function. For 4 inputs, gauss2mf shows the error distribution much closer to zero. The peak of the histogram curve of gauss2mf (i.e., 40 data) is higher than that of the other membership functions (i.e., 35 data). In addition, gauss2mf represents the minimum MSE (~ 0.0007). Although the best intelligence of the ANFIS is verified by 4 inputs and gauss2mf, the nanofluid temperature is considered as the fifth input as it is required for developing the pressure correlation. As Figs. 6a,b show, similar results to Fig. 5 are obtained and no changes in the histogram curves or in other words, in the intelligence of the ANFIS are found by 5 inputs.

**Figure 7:**
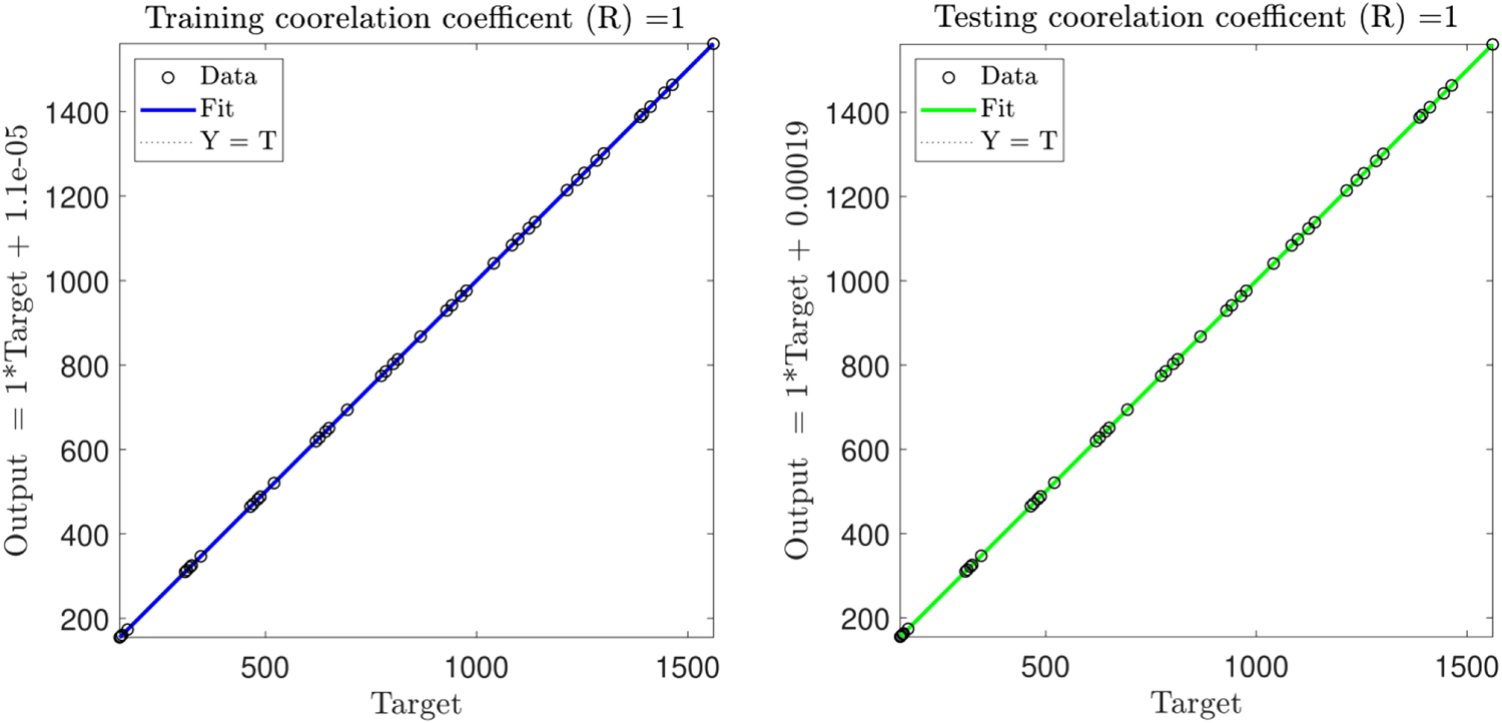
The plot of regression for the best intelligence. The number of inputs = 5. MF is guass2mf.

The intelligence of ANFIS is confirmed, as shown in Fig. 7, where the correlation coefficient (R) is about 1 for five inputs and the membership function of gauss2mf.

**Figure 8:**
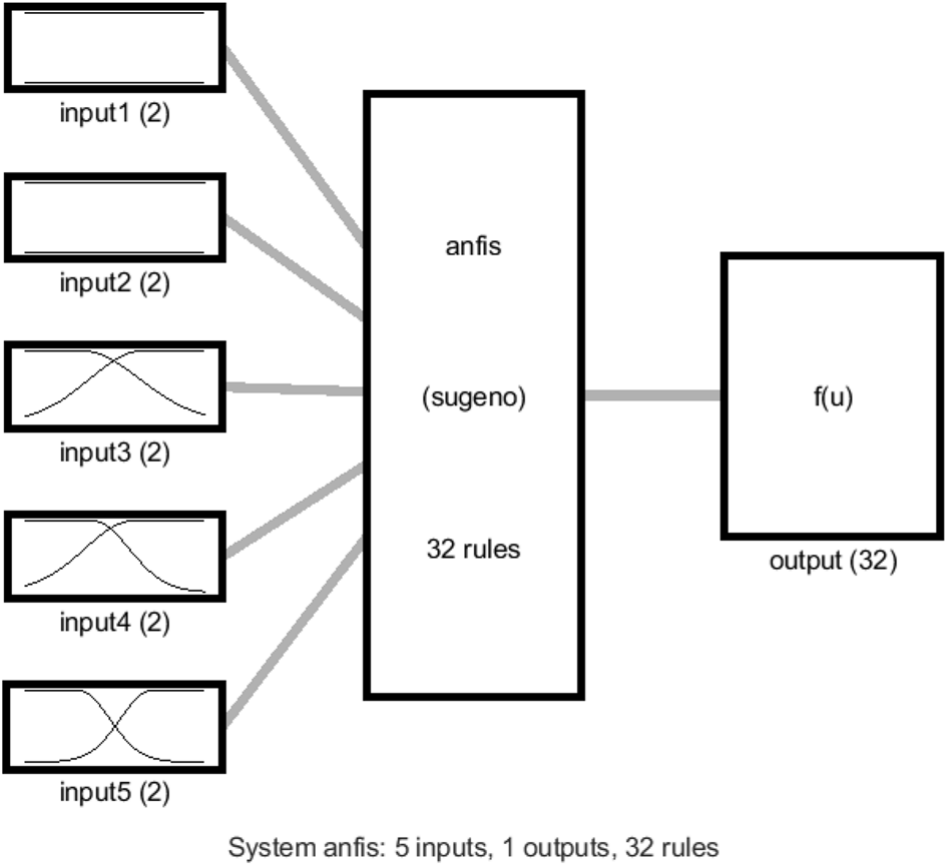
The FIS architecture for the best intelligence. The number of rules = 32.

**Figure 9:**
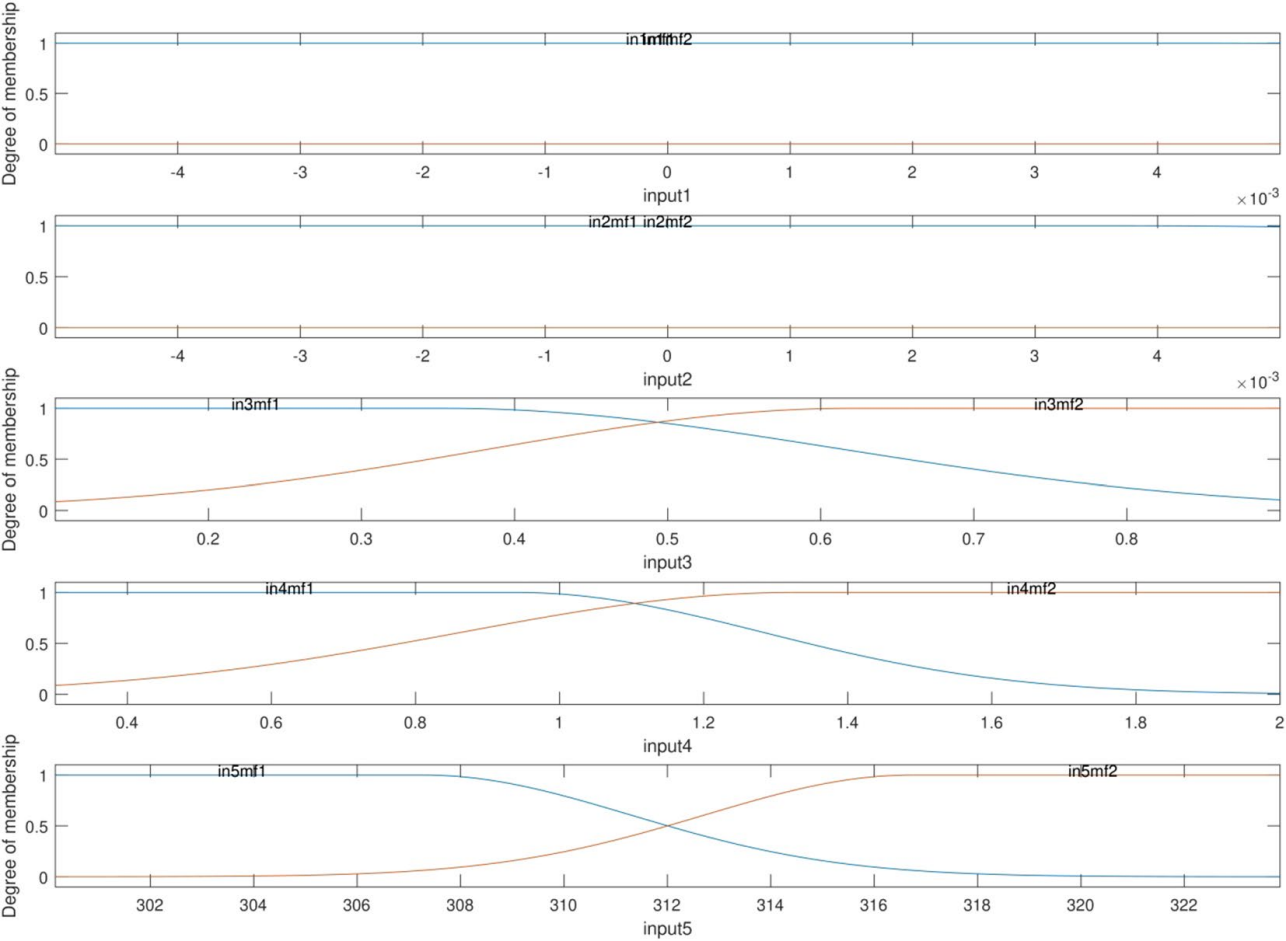
The degree of membership plots. The number of inputs = 5. MF is guass2mf.

According to Eq. (18), the pressure correlation is obtained once the Gaussian 2 function (µ) and the ANFIS factors (o, p, q, r, and s) are calculated. Table 4 represents the Gaussian 2 membership functions and their parameters (i.e., c1, σ1, c2, and σ2). After finding the required intelligence, the values of the Gaussian 2 parameters (Table 5) and the consequent parameters (Table 6) are found. As mentioned before and on the basis of Fig. 8, there are ten MF, and 32 rules and MF for the output and hidden layers. Figure 9 illustrates the membership functions versus each input. So, the horizontal axis shows the input domain, while the vertical axis illustrates the degree of each membership function. Afterward, the pressure of the nanofluid could be found in the pipe domain for each temperature and nanoparticle fraction. It means that there is no need for complex CFD calculations.

**Table 4:** The mathematical equation of Gaussian2 function used in this work^34^.

MF	Formula
Gauss2mf left Gaussian	${e}^{\frac{-{\left(x-{c}_{1}\right)}^{2}}{2{{\sigma }_{1}}^{2}}}$
Gauss2mf right Gaussian	1−${e}^{\frac{-{\left(x-{c}_{2}\right)}^{2}}{2{{\sigma }_{2}}^{2}}}$

**Table 5:** The input parameters of the used MF in this work.

Inputs	Input MFs	Type of MFs	σ1	σ2	c1	c2
First input	'in1mf1'	'gauss2mf'	0.001699	−0.008	0.007813	0.004424
	'in1mf2'	'gauss2mf'	− 0.00165	0.018832	0.001699	0.008
Second input	'in2mf1'	'gauss2mf'	0.001699	− 0.008	0.008636	0.003734
	'in2mf2'	'gauss2mf'	0.003895	0.022601	0.001699	0.008
Third input	'in3mf1'	'gauss2mf'	0.135891	− 0.14	0.256088	0.354452
	'in3mf2'	'gauss2mf'	0.234646	0.620765	0.135891	1.14
Fourth input	'in4mf1'	'gauss2mf'	0.288769	− 0.21	0.34276	0.940536
	'in4mf2'	'gauss2mf'	0.463091	1.325477	0.288769	2.51
Fifth input	'in5mf1'	'gauss2mf'	4.023589	293.056	4.023602	307.2682
	'in5mf2'	'gauss2mf'	4.023672	316.743	4.023589	330.9553

**Table 6:** The consequent parameters of ANFIS for prediction of the pressure.

Output MFs	Output MFs yype	n	o	p	q	r	s
'out1mf1'	'linear'	0.275497	0.82292	− 1532.57	34.17052	− 0.0002	1525.282
'out1mf2'	'linear'	0.52357	− 0.44146	− 1532.47	34.03126	− 0.00413	1526.564
'out1mf3'	'linear'	0.224109	0.988455	− 1734.84	25.32453	− 0.00102	1685.098
'out1mf4'	'linear'	− 0.91572	1.709538	− 1736.93	25.25651	0.003761	1683.852
'out1mf5'	'linear'	0.002649	− 0.43341	− 1534.15	1.164731	− 0.00089	1534.194
'out1mf6'	'linear'	− 0.28313	0.454	− 1534.1	1.375231	0.000408	1533.682
'out1mf7'	'linear'	− 0.15027	− 0.6456	− 1735.8	1.113383	0.004175	1732.314
'out1mf8'	'linear'	0.659498	− 0.64732	− 1737.69	1.248364	0.002532	1734.363
'out1mf9'	'linear'	− 0.00102	0.003052	− 0.75118	0.836047	4.339269	0.02237
'out1mf10'	'linear'	0.000449	0.000544	− 0.62938	0.586339	5.216454	0.039553
'out1mf11'	'linear'	− 0.0059	0.001832	− 0.81055	− 0.6955	5.137623	0.037629
'out1mf12'	'linear'	− 0.00043	0.000547	− 0.34753	− 0.53658	3.804881	0.020145
'out1mf13'	'linear'	− 0.00403	0.000384	0.69981	− 1.05822	0.61711	− 0.01135
'out1mf14'	'linear'	− 0.00417	0.000289	0.663309	− 1.86711	− 0.28804	0.001906
'out1mf15'	'linear'	− 6.74E−05	0.000251	0.838159	0.990119	− 0.04189	− 0.02188
'out1mf16'	'linear'	− 0.00322	3.46E−05	0.415818	1.835265	0.711474	0.030256
'out1mf17'	'linear'	− 8.31E−14	1.18E−14	− 5.45E−12	− 4.81E−12	− 5.38E−09	− 1.76E−11
'out1mf18'	'linear'	5.20E−14	5.01E−14	7.73E−12	1.89E−11	3.30E−09	1.03E−11
'out1mf19'	'linear'	− 1.72E−14	− 1.05E−14	− 3.08E−12	− 4.75E−12	− 1.11E−09	− 3.45E−12
'out1mf20'	'linear'	4.10E−14	2.27E−14	4.52E−12	2.07E−13	2.68E−09	8.34E−12
'out1mf21'	'linear'	− 1.49E−14	− 3.93E−15	2.25E−12	− 8.72E−12	− 9.73E−10	− 3.25E−12
'out1mf22'	'linear'	9.94E−14	3.70E−14	1.20E−11	2.86E−12	6.35E−09	2.01E−11
'out1mf23'	'linear'	− 4.60E−14	− 1.22E−14	− 6.35E−12	− 1.26E−11	− 3.09E−09	− 1.01E−11
'out1mf24'	'linear'	1.08E−13	1.55E−14	1.71E−11	4.34E−11	6.83E−09	2.16E−11
'out1mf25'	'linear'	− 3.22E−21	− 8.95E−22	− 9.30E−19	− 2.41E−19	− 2.33E−16	− 7.59E−19
'out1mf26'	'linear'	2.53E−20	5.01E−21	2.03E−18	4.96E−18	1.64E−15	5.14E−18
'out1mf27'	'linear'	− 4.61E−21	− 9.84E−22	− 9.51E−19	− 1.89E−18	− 3.13E−16	− 9.97E−19
'out1mf28'	'linear'	1.47E−20	2.40E−21	9.09E−19	1.86E−18	9.31E−16	2.91E−18
'out1mf29'	'linear'	− 6.97E−21	− 2.73E−21	− 5.37E−19	− 2.12E−18	− 4.74E−16	− 1.54E−18
'out1mf30'	'linear'	2.32E−20	4.68E−21	2.74E−18	2.11E−18	1.53E−15	4.79E−18
'out1mf31'	'linear'	− 1.21E−20	− 3.42E−21	− 1.59E−18	− 3.57E−18	− 8.19E−16	− 2.66E−18
'out1mf32'	'linear'	1.38E−20	2.45E−21	1.94E−18	4.38E−18	9.02E−16	2.83E−18

**Figure 10:**
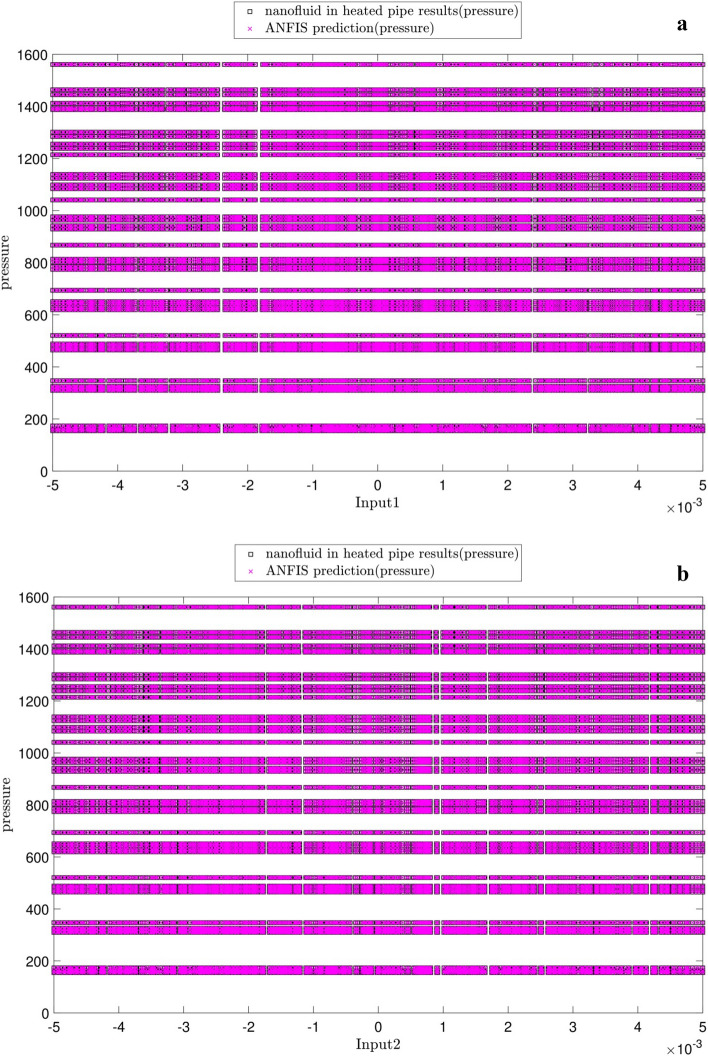

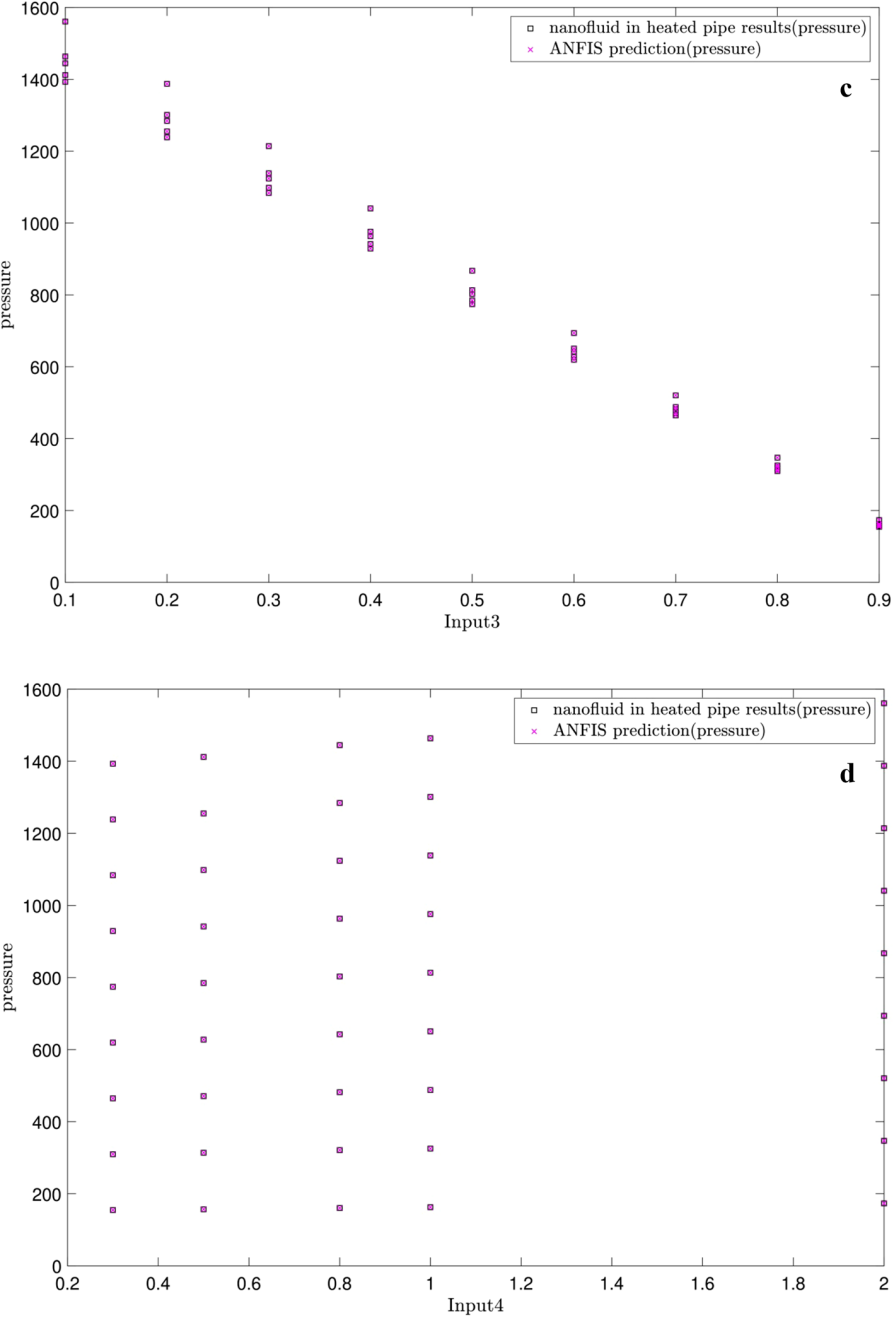

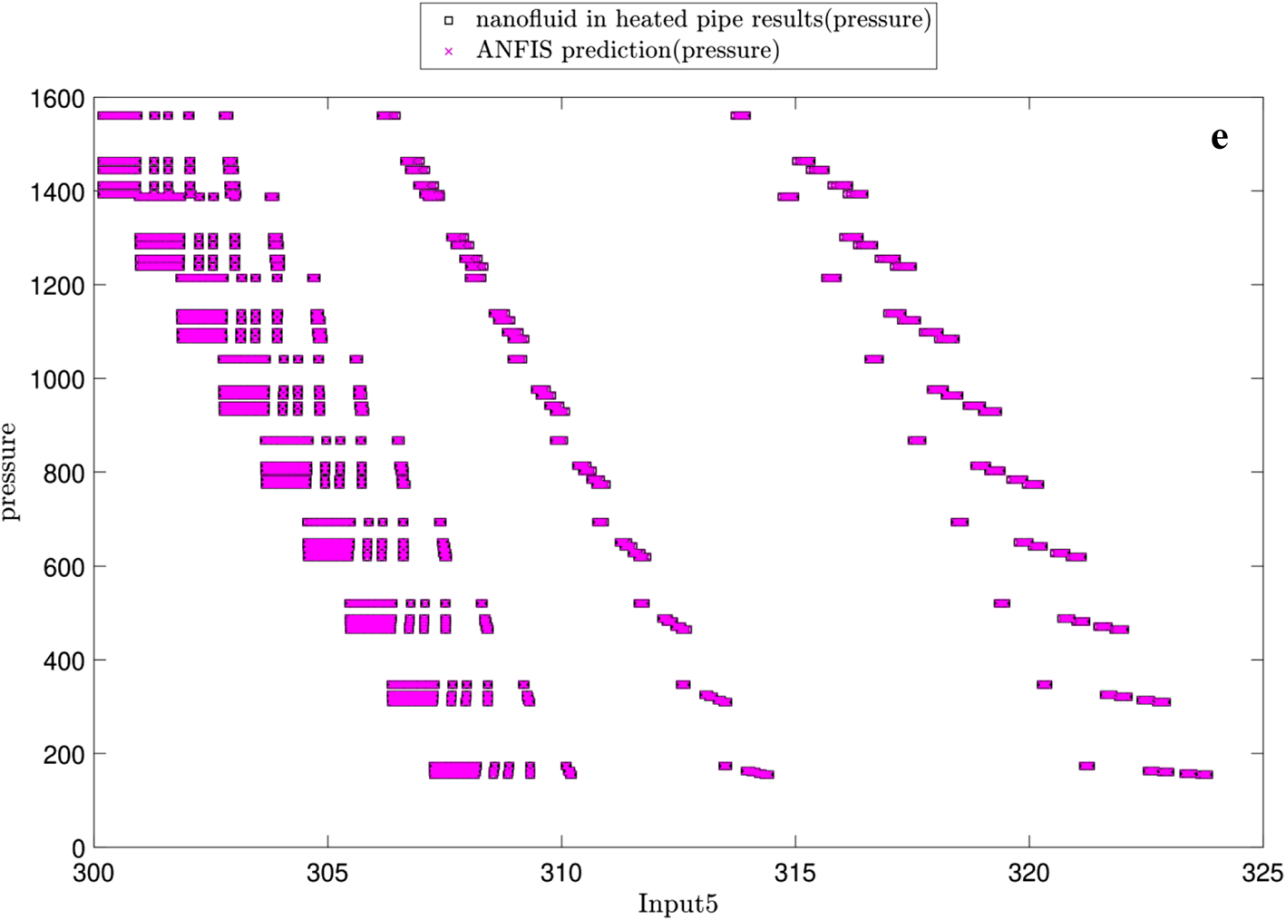
(**a**) Relation between pressure as output and x-direction as first input. (**b**) Relation between pressure as output and y-direction as second input. (**c**) Relation between pressure as output and z-direction as third input. (**d**) Relation between pressure as output and nanofluid fraction as fourth input. (**e**) Relation between pressure as output and temperature as fifth input.

Figure 10 illustrates the changes in the pressure versus each input. At first glance, there is a great agreement between the predicted pressures by both ANFIS and CFD. As be expected, no pressure changes are seen for the same cross-section planes (Figs. 10a,b). The pressure decreases in z-direction along the tube length (Fig. 10c). The pressure increases by the nanoparticle fraction (Fig. 10d) and decreases by the temperature (Fig. 10e).

**Figure 11:**
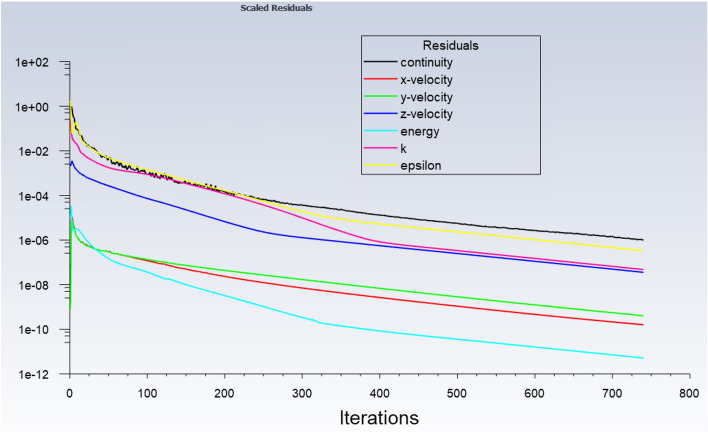
Scaled residual errors.

According to Fig. 11, the CFD solution was converged when the scaled residual of the algebraic equations of the continuity, the velocity components, the energy and the turbulent kinetic energy (*k*), and the turbulent dissipation rate (ε) would be less than 10^–6^. The performance of the CFD and the ANFIS are given in Tables 7 and 8, respectively, for a comparison between both methods in the case of the computational efforts. For the CFD modeling, the convergence requirements are satisfied for around 700 iterations in each simulation. Every simulation takes around 90–110 min. However, for the ANFIS method, the learning and the prediction times together for all types of the membership function is less than 369 s. It should be noted that the CFD modeling was done on a workstation computer, while the ANFIS method was run on a normal desktop. Table 9 illustrates the computer configurations using in this study. So, using the artificial intelligence approach in cooperation with the CFD approach could save time and expenses.

$$Pressure = \frac{{\mathop \sum \nolimits_{i = 1}^{2} \mathop \sum \nolimits_{j = 1}^{2} \mathop \sum \nolimits_{k = 1}^{2} \mathop \sum \nolimits_{l = 1}^{2} \mathop \sum \nolimits_{m = 1}^{2} \left( {\mu_{1i} \times \mu_{2j} \times \mu_{3k} \times \mu_{4l} \times \mu_{4m} } \right) \times \left( {n_{m} X \times o_{m} Y \times p_{m} Z \times q_{m} \varphi \times r_{m} T \times s_{m} } \right)}}{{\mathop \sum \nolimits_{i = 1}^{2} \mathop \sum \nolimits_{j = 1}^{2} \mathop \sum \nolimits_{k = 1}^{2} \mathop \sum \nolimits_{l = 1}^{2} \left( {\mu_{1i} \times \mu_{2j} \times \mu_{3k} \times \mu_{4l} \times \mu_{4m} } \right)}}$$

**Table 7:** CFD performance.

Scaled residual	Velocity components: 10^–6^ Energy: 10^–8^
Number of iterations	700–800
Time	90–110 min

**Table 8:** ANFIS performance.

Type of MFs	dsigmf	gauss2mf	gaussmf	gbellmf	psigmf
Number of inputs	5	5	5	5	5
Maximum of Iteration	60	60	60	60	60
Percentage of P	75	75	75	75	75
Clustering Type	Grid Partition	Grid Partition	Grid Partition	Grid Partition	Grid Partition
Learning time(s)	363.2	367.6	365.1	358.2	361.8
Prediction time(s)	1.48	1.51	1.41	1.55	1.47

**Table 9:** Computer configuration for ANFIS and CFD.

Processor using for CFD	Intel Xeon CPU E5-2685 v3 @ 2.60 GHz, 12 Cores
Processor using for ANFIS	Intel Core i5 CPU 650 @ 3.20 GHz, 3333 MHz, 2 Cores

In which:

*µ*_*1i* =_$$e^{{\frac{{ - \left( {x - c_{i} } \right)^{2} }}{{2\sigma^{2} }}}}$$, *µ*_*2j* =_$$e^{{\frac{{ - \left( {x - c_{j} } \right)^{2} }}{{2\sigma^{2} }}}}$$, *µ*_*3k* =_$$e^{{\frac{{ - \left( {x - c_{k} } \right)^{2} }}{{2\sigma^{2} }}}}$$, *µ*_*4l* =_$$e^{{\frac{{ - \left( {x - c_{l} } \right)^{2} }}{{2\sigma^{2} }}}}$$, and *µ*_*5m* =_$$e^{{\frac{{ - \left( {x - c_{m} } \right)^{2} }}{{2\sigma^{2} }}}}$$.

## Conclusions

The nanofluid flow characteristics such as thermal conductivity and dynamic viscosity are influenced by the nanoparticles fraction in the whole volume of the base fluids. The pressure is one of the important flow parameters for pressure drop determination. The nanoparticle volume fraction, in turn, could highlight the impact of thermal conductivity on the temperature distribution. The temperature also affects the viscosity, and it is followed by the changes in the pressure. So, the pressure could be a function of the nanoparticle fraction and the temperature of the fluid flow. The computational fluid dynamics (CFD) approach is able to predict such changes. But this approach faces two main weak points. The former is the inability of the CFD in finding the connection of the pressure to the nanoparticle fraction and the temperature. The latter is related to the efforts and expenses relating to the simulation time and infrastructures.

The idea of this study was to improve the CFD shortages by establishing an auxiliary method. Thus, the artificial intelligence algorithm was introduced and investigated for such purposes. The anticipation of Cu/water in a heated wall pipe was considered as a sample case. Primarily, the nanofluid was simulated using the finite volume method of the CFD tool. The CFD results were verified with the experiment from the literature. Then, the CFD results were learned by ANFIS as the artificial intelligence algorithm. The ANFIS could capture the pattern of the pressure changes of the nanofluid by the position (i.e., x, y, and z), the particle content, and the temperature.

The intelligence of the ANFIS method was examined by different inputs and different membership function types (i.e., gbellmf, gaussmf, gauss2mf, dsigmf, psigmf). The ANFIS was compared with the CFD regarding the accuracy of the prediction and performance. The following results have been achieved from this study:

- For 5 inputs and the membership function of the Gauss2mf, the histogram graphs of the sensitivity analysis showed the error distribution and the correlation coefficient close to 0 and 1, respectively.
- The artificial intelligence of ANFIS could predict the pressure with the same values of the CFD prediction.
- The artificial intelligence of ANFIS could find the relation of the nanofluid pressure to the nanoparticle fraction and the temperature. The CFD simulation could be replaced by such a correlation.
- Every CFD simulation took around 90–110 min. However, for the ANFIS method, the learning and the prediction times together for all types of the membership function was less than 369 s. The CFD modeling was done on a workstation computer, while the ANFIS method was run on a normal desktop.
- So, using the artificial intelligence approach in cooperation with the CFD approach could save time and expenses.

## List of symbols

C_p_: Specific heat capacity of fluid/solid (J/kg K)
d_p_: Diameter of particle (m)
FVT: Finite volume technique
k: Thermal conductivity (W/m K)
*k*: Turbulence kinetic energy (m^2^/s^2^)
*K*_*B*_: Boltzmann constant
*p*: Pressure (Pa)
Re: Reynolds number $(\frac{{\rho V_{in} D}}{\mu })$
T: Temperature (K)
UDF: User defined function
V: Velocity (m/s)

## Greek letters

ε: Dissipation rate of turbulence kinetic energy (m^2^/s^3^)
µ: Dynamic viscosity (kg/m s)
ρ: Density (kg/m^3^)
φ: Particle volume fraction
τ: Shear stress (N/m^2^)

## Subscriptions

bf: Base fluid
eff: Effective
f: Fluid
p: Particle phase
l: Liquid phase
